# Synergistic Effect of Perineural Dexamethasone and Dexmedetomidine (Dex-Dex) in Extending the Analgesic Duration of Pectoral Type I and II Blocks

**DOI:** 10.7759/cureus.10703

**Published:** 2020-09-29

**Authors:** Robert P Zusman, Ivan Urits, Alan D Kaye, Omar Viswanath, Jonathan Eskander

**Affiliations:** 1 Department of Anesthesiology, Mount Sinai Medical Center, Miami Beach, USA; 2 Department of Anesthesia, Critical Care and Pain Medicine, Beth Israel Deaconess Medical Center, Harvard Medical School, Boston, USA; 3 Department of Anesthesiology, Louisiana State University Shreveport, Shreveport, USA; 4 Department of Pain Management, Valley Pain Consultants - Envision Physician Services, Phoenix, USA; 5 Department of Anesthesiology and Pain Medicine, Portsmouth Anesthesia Associates, Portsmouth, USA

**Keywords:** regional anesthesiology, perioperative pain management, pain, acute pain, pain management, chronic and acute pain management, regional blocks, dex-dex

## Abstract

Pectoral type I and II (Pecs I and II) blocks are regional anesthesia methods that have shown to decrease postoperative analgesia after breast surgery. Typically, these blocks consist only of a local anesthetic. We performed preoperative Pecs I and II blocks in a female patient undergoing surgical excision and biopsy of a breast mass. The anesthetic consisted of ropivacaine, dexmedetomidine, and dexamethasone (Dex-Dex). The patient experienced an extended postoperative pain relief period. She did not require any opiates postoperatively. Adding dexmedetomidine and dexamethasone to a local anesthetic for peripheral nerve blocks seems to have a synergistic effect and can extend the duration of pain relief. This combination has the potential to decrease postoperative opiate requirements for analgesia. Further studies need to be conducted to further determine the safety and efficacy of the Dex-Dex block.

## Introduction

Pectoral type I and II (Pecs I and II) blocks are safe and effective methods of providing postoperative analgesia for patients undergoing mastectomy [1]. These blocks have been shown to decrease postoperative opiate consumption and decrease pain levels compared to patients solely undergoing general anesthesia [2]. Adding dexamethasone to the Pecs blocks has shown to prolong postoperative analgesia [3]. Similar results were observed when adding dexmedetomidine [4]. Despite these findings, there is limited research on using the combination of dexamethasone and dexmedetomidine (Dex-Dex) on extending postoperative analgesia.

## Case presentation

**Figure 1:**
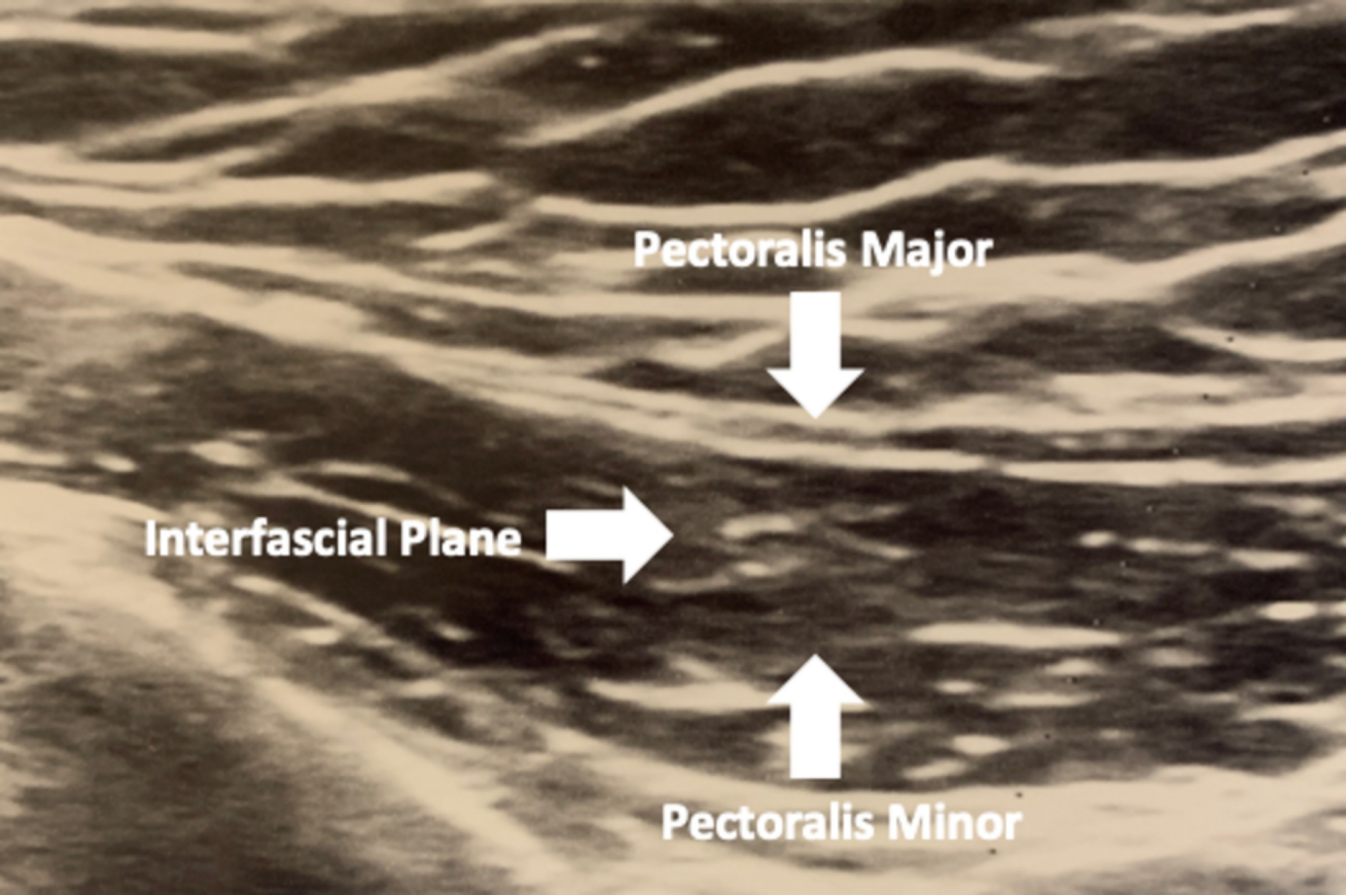
Antero-posterior ultrasound view of the pectoralis major and pectoralis minor muscles.

We performed a preoperative ultrasound-guided Pecs I and II blocks for surgical excision and biopsy of a cystic mass of the left breast located on the medial aspect of the breast 2 cm from the sternal border. Our patient was a 68-year-old African American female with a past history remarkable for dyslipidemia, mitral valve prolapse, partial hysterectomy, and appendectomy. She was requiring oxycodone-acetaminophen as needed for pain. Patient consent to publish this case was obtained. After induction of anesthesia, we performed Pecs I and II blocks for postoperative pain control. The patient was supine on an operating table, with her left arm placed at her side. A low-frequency ultrasound transducer was placed over the lateral aspect of the anterior chest wall and sterile coupling gel was applied. The thoracoacromial artery, pectoralis major, pectoralis minor, serratus anterior, and intercostal muscles were identified (Figure 1). An echogenic block needle was advanced in-plane coming to rest in between the pectoralis major and pectoralis minor. The anesthetic consisted of 30 mL of 0.2% ropivacaine, dexmedetomidine 25 mcg, and dexamethasone 4 mg. Hydrodissection was performed between the pectoralis major and the pectoralis minor, and 15 mL of the anesthetic was administered into the area. The needle was then relocated laterally and deeper, coming to rest between the pectoralis minor and serratus anterior muscles, and an additional 15 mL of the anesthetic was administered.

Her sensory nerve block lasted for seven days, and the patient reported no postoperative pain 14 days later. Postoperatively, she required no opioid medications and her pain was adequately managed with acetaminophen 1,000 mg by mouth three times daily. In this patient, the Dex-Dex combination demonstrated extended postoperative pain relief. The patient experienced no adverse effects. Although both dexamethasone and dexmedetomidine have been extensively studied separately, there is little data on the use of these two agents together.

## Discussion

Hassan et al. compared the effects of the Dex-Dex combination to using each agent by itself in combination with bupivacaine in caudal blocks for pediatric hypospadias repair. Primary outcomes of the study were pain scores and time to analgesia request. Patients who received the Dex-Dex combination had significantly decreased pain scores up to six hours after surgery compared to those who received only dexamethasone or only dexmedetomidine. Time to first use of analgesia was four hours longer than the dexmedetomidine group and six hours longer than the dexamethasone group, respectively [5]. Another study observed the effect of the Dex-Dex block in intercostal nerve blocks for thoracoscopic pneumonectomy. Zhang et al. found that patients who received the Dex-Dex combination had approximately 3.5 more hours of postoperative pain relief and required significantly less fentanyl after surgery when compared to dexmedetomidine and dexamethasone groups [6]. Additionally, other case reports have demonstrated a prolonged analgesic effect when using the Dex-Dex combination in interscalene and transversus abdominis plane blocks [7,8].

Dexamethasone is commonly used in clinical practice as an adjuvant for regional anesthesia. There has been conflicting evidence as to whether or not it prolongs postoperative analgesia. The mechanism of action is not fully understood, but the belief is that dexamethasone has an anti-inflammatory effect when administered perineurally [9]. In animal studies, perineural administration of local anesthetic with adjuvant dexmedetomidine has been shown to increase the duration of analgesia by blocking hyperpolarized cation channels [10]. Huang et al. demonstrated the anti-inflammatory effects of dexmedotomidine in rats. When dexmedetomidine is injected into the sciatic nerve of rats at high doses, it inhibits NF-κB and reduces proinflammatory cytokine production [11]. Many studies have demonstrated greater postoperative pain relief when using dexmedetomidine in peripheral nerve blocks, but there is a slightly increased risk of adverse effects such as bradycardia and hypotension [12].

## Conclusions

This case exhibits a possible additive and/or synergistic mechanism of dexamethasone and dexmedetomidine when used together in a peripheral nerve block. More studies need to be conducted to evaluate the safety and efficacy of this combination. The Pecs I and II blocks have been shown to decrease postoperative pain and opiate consumption. Adding Dex-Dex could potentially extend the postoperative analgesia period of the Pecs I and II blocks. This treatment modality could be an advancement in regional anesthesia and provide patients with superior pain control and reduced postoperative opiate use.
